# The impact of environmental factors in pre-hospital thermistor-based tympanic temperature measurement: a pilot field study

**DOI:** 10.1186/s13049-015-0148-5

**Published:** 2015-09-24

**Authors:** Sven Christjar Skaiaa, Guttorm Brattebø, Jörg Aßmus, Øyvind Thomassen

**Affiliations:** Department of Anaesthesia & Intensive Care, Oslo University Hospital, N 0424 Oslo, Norway; Department of Anaesthesia & Intensive Care, Haukeland University Hospital, N 5021 Bergen, Norway; Centre for Clinical Research, Haukeland University Hospital, N 5021 Bergen, Norway

## Abstract

**Background:**

Few pre-hospital services have the possibility to accurately measure core temperature (*T*_*core*_). Non-invasive estimation of *T*_*core*_ will improve pre-hospital decision-making regarding the triage and management of hypothermic patients. Thermistor-based tympanic temperature (*T*_*tymp*_) correlates well with *T*_*core*_ in controlled studies; however, little is known about the feasibility of using *T*_*tymp*_ under field conditions. This study assessed the impact of pre-hospital environmental factors on the accuracy of *T*_*tymp*_. Deep rectal temperature (*T*_*rect*_) was used as a substitute for *T*_*core*_.

**Methods:**

Normothermic volunteers (*n* = 13) were exposed to four simulated field conditions producing local cooling of the head and ear canal. After exposure, *T*_*tymp*_ was recorded every 15 s for 10 min and compared with *T*_*rect*_. Descriptive analysis and Bland-Altman plots were used to assess agreement.

**Results:**

Immediately after exposure mean *T*_*tymp*_ was low, but increased rapidly and reached an apparent steady state after 3–5 min. After 5 and 10 min, the mean temperature difference (∆*T*_*rect-tymp*_) ranged from 1.5–3.2 °C (SD = 0.5) and 1.2–2.0 °C, respectively. *T*_*rect*_ remained unchanged throughout the study period.

**Conclusions:**

After surface cooling of head and neck, *T*_*tymp*_ did not accurately reflect core temperature within the first 10 min of measurement. The variation of ∆*T*_*rect-tymp*_ was low after 10 min, regardless of the initial degree of cooling. With the risk of over-triage, *T*_*tymp*_ may at this point provide an indication of *T*_*core*_ and also exhibit a trend.

**Trial registration:**

ClinicalTrials.gov: NCT02274597

**Electronic supplementary material:**

The online version of this article (doi:10.1186/s13049-015-0148-5) contains supplementary material, which is available to authorized users.

## Background

A core temperature (*T*_*core*_) < 30 °C prior to cerebral hypoxia (i.e. avalanche, drowning) or out-of-hospital-cardiac-arrest (OHCA) is associated with increased chances of survival and a neurological favourable outcome [1–8]. For most other patients, accidental hypothermia is associated with increased morbidity and mortality and should be treated aggressively [9–16]. Early measurement of *T*_*core*_ is important to diagnose and treat hypothermia and will, for OHCA, aid the decision to either continue resuscitation to hospital or declare the patient dead on-scene [17, 18]. Temperature measurement becomes increasingly useful in rural and wilderness areas with long transports and in mass casualty incidents when resources are limited. Unfortunately*, T*_*core*_ is infrequently evaluated in the field, and many pre-hospital services do not have adequate equipment to accurately diagnose hypothermia [10, 19, 20]. Ideally, *T*_*core*_ is measured in the distal third of the oesophagus [21, 22], but requires the patient to be sedated or intubated. Common non-invasive techniques include the ear canal, nasopharynx and rectum. Historically, one of the most accepted techniques for field use is the tympanic thermistor-based temperature (*T*_*tymp*_*)*. Studies and case reports have shown that *T*_*tymp*_ accurately reflects brain temperature; therefore, it has been accepted as a good non-invasive estimation of *T*_*core*_ [23–25]. Several factors may influence the accuracy of *T*_*tymp*_, and *T*_*tymp*_ has been shown to decrease in cold ambient temperatures [26]. Other mechanisms that might influence accuracy include wind and direct cooling of the external auditory canal, as well as equipment failure in cold or wet conditions. The aim of this study was to investigate the impact of environmental factors on the accuracy of *T*_*tymp*_ under field conditions.

## Methods

### Study design

In this observational study we compared *T*_*tymp*_ with deep rectal temperature (*T*_*rect*_) in healthy and normothermic volunteers during four separate exposure scenarios designed to simulate probable real-life situations. Because *T*_*rect*_ accurately reflects *T*_*core*_ in normothermic patients in a stable environment [22, 27], it was defined as *T*_*core*_ in this study.

To ensure normothermia, the volunteers were outdoors only during the scenarios. They were placed sitting on a 14-mm sleeping mat (Mammut Bamse Extreme, Mammut Sports Group, Switzerland) in a shady area to avoid direct sunlight. All volunteers were dressed similarly: in a base layer of woollen thermal underwear, with an outer shell of an expedition down jacket with a hood and Gore-Tex® pants. A woollen hat and mittens were used for insulation of head and hands. The lower body was covered with a thermal rescue bag (Fjellduken Thermo Hunter, Jerven AS, Norway).

Each volunteer served as his or her own control. Each exposure scenario was analysed as an entity to ensure as similar ambient conditions as possible, but also to allow a minimum of 100 min of indoor rest between each scenario to avoid carry-over effects.

*T*_*rect*_ was measured using a standard multi-purpose probe (Mon-a-Therm, General purpose Temperature Probe 12Fr/Ch, Covidien, Ireland) attached to an external monitor (Propaq Encore 206EL, ZOLL Medical Corporation, MA, USA). The probe was placed 20 cm into the rectum and taped to the skin.

A thermistor-based thermometer (Métraux®, Crissier, Switzerland) was used to measure *T*_*tymp*_. The thermometers accuracy was verified at Oslo University Hospital, Dep. of Medical Technology. In the temperature range of 22.4–38.0 °C it was found to display an accuracy of ± 0.2 °C (OUS Rikshospitalet, MTV, ref.no. 942252, Oct. 2013). The sensor consists of two thin and malleable plastic-covered metal wires integrated into a soft rubber earplug. After each exposure scenario the ear sensor was inserted into the external auditory canal. The earplug was securely taped to the external ear to avoid dislocation during the tests. We were able to visually read *T*_*tymp*_ in real time from the instrument’s display. Otoscopy was performed before the first insertion and after the completion of all tests.

Ambient temperature was measured with an external monitor (Propaq Encore 206EL, ZOLL Medical Corporation, MA, USA). Wind speed was measured using a Silva wind gauge instrument (Silva ADC Wind, Silva Sweden AB, Sweden).

Each volunteers was exposed to all of the four exposure scenarios in the following order:

- Ambient air without local insulation/Wind (Ins -). Simulation of a normal outdoor setting. The volunteer was exposed to ambient air and wind without protection of the head for 10 min before placement of the ear sensor. No additional insulation of the head/ear was applied during data collection.
- Ambient air with local insulation/Wind (Ins +). Exposure identical to scenario A., but after placement of the ear sensor the head was covered with a woollen hat and the down jacket hood was engaged.
- Snow in ear canal/Snow (Ins +). Simulation of a victim buried in an avalanche. Snow was manually packed densely into the external auditory canal and left for 4 min. After 2 min, any melted snow was replaced with fresh snow. After 4 min, the remaining snow was removed, and the external auditory canal was dried for 3–4 s with a towel before placement of the ear sensor. Finally, the head was covered with a woollen hat and the down jacket hood was engaged.
- Icy water in ear canal/Water (Ins +). Simulation of a victim immersed in cold water. The volunteer submerged his/her entire head and neck in a 12-liter bucket of icy water (1-4 °C) for 5–10 s. Submersion was conducted three consecutive times, with a 1-min rest between submersion periods. Immediately after the final submersion, the external auditory canal was dried for 3–4 s with a towel before placement of the ear sensor. Finally, the head was covered with a woollen hat and the down jacket hood was engaged.

### Study volunteers

Inclusion required that volunteers be older than 18 years of age, non-smokers, and healthy individuals. Volunteers were excluded if otoscopy revealed pathology in the external auditory canal.

### Data collection

The study took place at 860 m above sea level during 2 days in May 2014. *T*_*rect*_ and *T*_*tymp*_ baseline was initially measured after a minimum of 30 min at rest indoors in a median ambient temperature of 20.5 °C (19.5–21.1). The volunteers were then moved outdoors in random pairs. *T*_*rect*_*w*as measured before each scenario (baseline) and then every 15 s during the first 20 scenarios. As *T*_*rect*_ remained unchanged (≤0.1 °C), we then reduced the measuring interval to every 10 min (before, during and after each scenario). *T*_*tymp*_ was recorded every 15 s for 10 min after each exposure scenario. *T*_*tymp*_ baseline was not repeated before each scenario to avoid pre-warming of the sensor. Ambient temperature and wind speed were recorded every 30 min throughout both study days.

### Statistical analysis

We used descriptive analysis (median, minimum, maximum, percentiles, and confidence intervals) and graphical methods to characterize the temperature trajectories. The comparison of the measurement methods was done with Bland-Altman plots with 95 % limits of agreement. P-values less than 0.05 were considered significant. All computation and graphics were completed using Matlab 7.10 (Mathworks Inc).

### Ethical considerations

This study was approved by the Regional Ethics Committee (REK sør-øst, ref 2014/108).

## Results

A total of 13 volunteers (nine women) were included. Written informed consent was obtained. Median age was 25 years (range 20–39), median BMI 22.6 kg/m^2^ (range 20.2–27.5). Median *T*_*rect*_ at baseline was 37.4 °C (range 36.8–37.6). The variation of *T*_*rect*_ between baseline, before, during and after each scenario was ≤ 0.1 °C and hence considered constant. Median *T*_*tymp*_ at baseline was 36.5 °C (range 36.0–37.5) and significantly lower than *T*_*rect*_ (*P* < 0.001). Respectively, the median ambient temperature and wind speed during both study days were 1.6 °C (range 0.5–5.0) and 5.5 m/s (range 2.7–10.0).

**Fig. 1:**
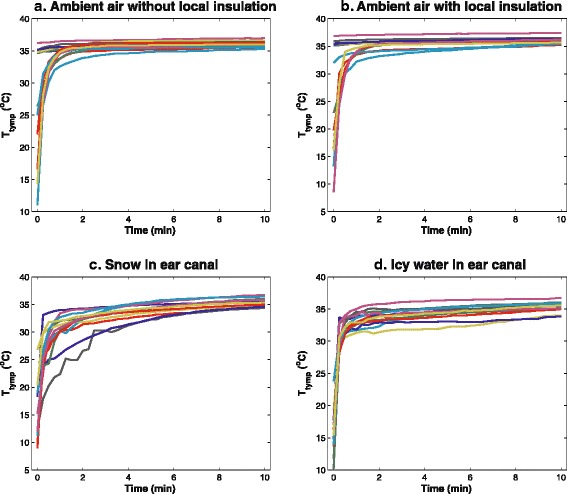
(Top left panel): Change in *T*_*tymp*_ after exposure in scenario **a**. (Top right panel): Change in *T*_*tymp*_ after exposure in scenario **b**. (Bottom left panel): Change in *T*_*tymp*_ after exposure in scenario **c**. (Bottom right panel): Change in *T*_*tymp*_ after exposure in scenario **d**. Time = 0 is the first measurement after exposure was terminated

*T*_*tymp*_ was initially low after the exposure was terminated; it increased rapidly for approximately 1 min, and at a much slower rate thereafter (Fig. 1). *T*_*tymp*_ continued to increase throughout the 10-min study period, although at a minimal rate after 3–5 min, reaching an apparent steady state.

**Fig. 2:**
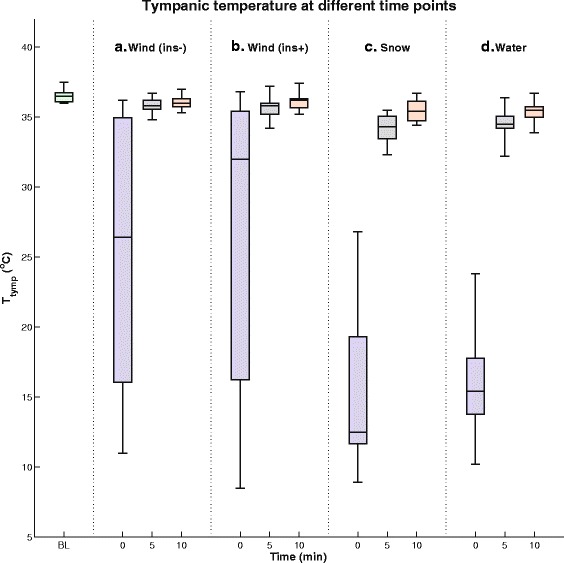
(Far left column): Box plot of *T*_*tymp*_ at baseline (BL). (Column a): Box plots of *T*_*tymp*_ at 0, 5, and 10 min for scenario **a**. (Column b): Box plots of *T*_*tymp*_ at 0, 5, and 10 min for scenario **b**. (Column c): Box plots of *T*_*tymp*_ at 0, 5, and 10 min for scenario **c**. (Column d): Box plots of *T*_*tymp*_ at 0, 5, and 10 min for scenario **d**

The spread of *T*_*tymp*_ at baseline and after 0, 5, and 10 min, is schematized in Fig. 2. *T*_*tymp*_ was significantly lower than baseline values at 0 and 5 min for all tests (*P* < 0.006, Additional file 1). The spreading decreased markedly after 5 min. After 10 min the median *T*_*tymp*_ neared baseline, and scenario B (ambient air with insulation) did at this point not demonstrate a significant difference (P > 0.07).

**Fig. 3:**
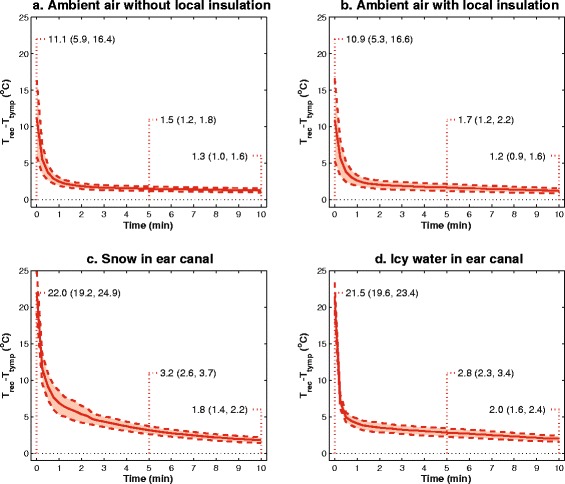
(Top left panel): Mean time-dependent ∆*T*_*rect-tymp*_ with 95 % confidence intervals for scenario **a**. (Top right panel): Mean time-dependent ∆*T*_*rect-tymp*_ with 95 % confidence intervals for scenario **b**. (Bottom left panel): Mean time-dependent ∆*T*_*rect-tymp*_ with 95 % confidence intervals for scenario **c**. (Bottom right panel): Mean time-dependent ∆*T*_*rect-tymp*_ with 95 % confidence intervals scenario **d**. Numeric values are presented at 0, 5 and 10 min

The time-dependent mean differences between *T*_*rect*_ and *T*_*tymp*_, with 95 % confidence intervals, are presented in Fig. 3. The mean difference was markedly reduced for all scenarios after 5 min, and continued to decrease at a slower rate throughout the study period. Snow in the ear canal provided the largest difference with mean ∆*T*_*rect-tymp*_ = 22.0 °C at 0 min, 3.2 °C at 5 min and 1.8 °C after 10 min.

**Fig. 4:**
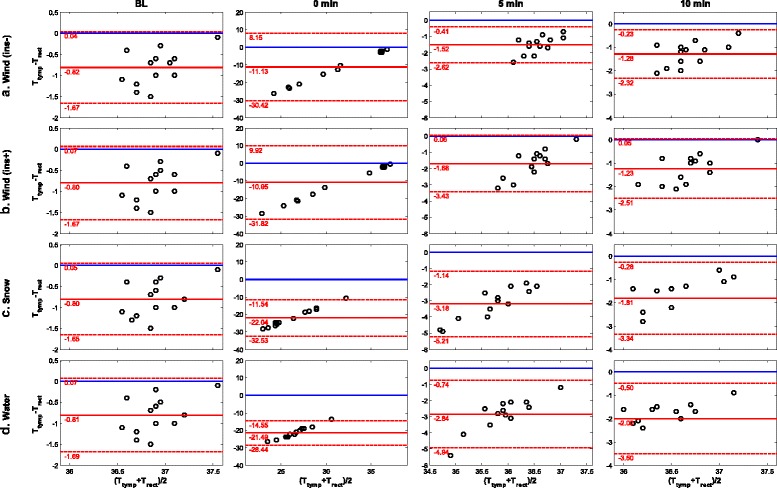
(Row a): Bland-Altman plots with mean and 95 % limits of agreement for scenario **a** at baseline (BL), 0, 5 and 10 min. (Row b): Bland-Altman plots with mean and 95 % limits of agreement for scenario **b** at baseline (BL), 0, 5 and 10 min. (Row c): Bland-Altman plots with mean and 95 % limits of agreement for scenario **c** at baseline (BL), 0, 5 and 10 min. (Row d): Bland-Altman plots with mean and 95 % limits of agreement for scenario **d** at baseline (BL), 0, 5 and 10 min

Bland-Altman plots were used to assess agreement between *T*_*rect*_ and *T*_*core*_ (Fig. 4). At baseline we observed a mean difference of 0.8 °C for all scenarios (*P* < 0.001). At 0 min an outlier is observed in scenarios A and B (recognisable as parallel lines in Fig. 1), probably representing a delay in data registration. During the 10 min measurement phase we observed a linear decrease in both the mean difference and range of agreement. After 10 min the mean difference ranged from 1.2 to 2.0 °C for scenarios B and D, respectively (*P* < 0.001). The concordance correlations showed a similar pattern, but were very low (≤0.063, Additional file 2).

## Discussion

### Overview

The first obtained *T*_*tymp*_ was very low compared to *T*_*rect*_ for all exposure scenarios. In correlation with previous studies [23], *T*_*tymp*_ then increased rapidly to an apparent steady state within a few minutes. After 5 and 10 min, mean ∆*T*_*rect-tymp*_ varied up to 3.2 and 2.0 °C, respectively. The thermometer used in this study did not accurately estimate the core body temperature*,* neither at baseline nor throughout the study. Rather than providing a true value, *T*_*tymp*_ gave an increasingly stable indication of *T*_*core*_, as confirmed in the Bland-Altman plots. The almost constant *T*_*rect*_, leading to Bland-Altman plots dominated by *T*_*tymp*_, caused the linear trend observed in the plots. This effect increased with the variation of *T*_*tymp*_, i.e. it was clearest visible directly after cooling and weakened with the reduction of the variation of *T*_*tymp*_ over time. The extremely low concordance correlations may be caused by the low variation in *T*_*rect*_ as well, since all correlation type analysis depend on the range of the considered variables.

Non-invasive thermometry will normally be utilized when the patient is not intubated or in the absence of invasive *T*_*core*_ devices. *T*_*tymp*_ will in these situations give an increasingly stable indication of *T*_*core*_, with the risk of modest over-triage. Drying the ear canal thoroughly and leaving the ear sensor in place for a minimum of 10 min will reduce this risk. In addition, in a field situation one can expect *T*_*core*_ to decrease further during on-site management and transport (with the possible exception of a well-circulated patient with intact shivering capacity that is re-warmed en-route). This reduces the significance of an initial over-triage for especially patients in moderate (*T*_*core*_ 28–32 °C) and deep (*T*_*core*_ <28 °C) hypothermia. The low variation after 10 min indicates that measurement of *T*_*tymp*_ may enable the health provider to monitor the *T*_*core*_ trend during treatment and transport.

Insulation of the head and neck with a woollen hat combined with a thick down jacket hood did not significantly improve accuracy. Possible explanations for this include cold equipment or insufficient ear padding, which could allow for heat loss through convection and evaporation. More likely, the ambient temperature might not have been cold enough to reveal a true benefit.

### Limitations

When exposed to rapid temperature changes, *T*_*rect*_ can lag behind *T*_*core*_. To avoid such changes, and to ensure normothermia, we insulated the participants well and reduced outdoor exposure times to a minimum. Deep *T*_*rect*_ remained stable during the scenarios and was therefore considered an accurate estimation of *T*_*core*_ in this study.

We did not repeat baseline *T*_*tymp*_ before exposure, as this would imply warming of the thermometer just prior to the post-exposure data collection and create a carry-over effect. As T_*rect*_ demonstrated stable core temperatures throughout the study we hypothesized that also T_*tymp*_ baseline would not change significantly after 100 min of indoor rest. However, a systematic bias cannot be excluded.

Despite standardization, a field study may be biased by changes in temperature and wind. After the volunteers were exposed to snow and icy water, all visible snow and water was removed from the ear before placing the temperature probe; however, the amount of residual snow or water cannot be estimated. Each of the exposure scenarios reflected a real-life situation. Although we did not measure the time used to attach and fix the thermometer, in most cases the first reading was less than 20 s after the cooling had stopped. Time is often a limiting factor in the clinical setting, and time spent to obtain access to a suitable temperature measurement site is reportedly one of several factors that contribute to low compliance in the field [20].

### Clinical implications and conclusions

In the field, a thermistor-based *T*_*tymp*_ will initially display very low values, with an apparent steady state to be expected after 3–5 min. *T*_*tymp*_ will however continue to increase after 10 min, and may at this point overestimate the degree of hypothermia up to a median of 2.0 °C in an ear canal cooled from snow or water. The tympanic probe should therefore be left in place in a dry ear canal for more than 10 min before estimating *T*_*core*_ as a basis for decision-making. The variation of *T*_*tymp*_ after 5 and 10 min was low in this study, indicating that *T*_*tymp,*_ in a non-OHCA setting, may be used with reasonable accuracy to exhibit the temperature trend during management and transport.

Future research should focus on developing non-invasive thermometer devices designed for rapid placement, better insulation against the environment, and improved accuracy.

## Additional files

Additional file 1:
**Signed rank test for ΔT** ***tymp*** **– T** ***tympbaseline*** **at 0, 5 and 10 min.** (PDF 63 kb) Additional file 2:
**Concordance correlation coefficients for Δ** ***Trect-tymp.*** (PDF 53 kb)
