# Supplementary material for: The impact of environmental factors in pre-hospital thermistor-based tympanic temperature measurement: a pilot field study
Source: Scand J Trauma Resusc Emerg Med. 2015 Sep 24;23:72. doi: 10.1186/s13049-015-0148-5 (PMC4581419; doi:10.1186/s13049-015-0148-5)
Supplement: Additional file 1: — Signed rank test for ΔT tymp – T tympbaseline at 0, 5 and 10 min. (PDF 63 kb) [file 13049_2015_148_MOESM1_ESM.pdf]

Additional file 1

Signed rank test for $\Delta T_{tymp} - T_{tympbaseline}$ at 0, 5 and 10 min

	**Baseline**	**0 min**		**5 min**		**10 min**	
	Median [Range]	Median [Range]	p-val	Median [Range]	p-val	Median [Range]	p-val
**A. Wind (Ins -)**	36.5 [36.0, 37.5]	26.4 [11.0, 36.2]	0.000	35.8 [34.8, 36.7]	0.006	36.0 [35.3, 37.0]	0.021
**B. Wind (Ins +)**	36.5 [36.0, 37.5]	32.0 [8.5, 36.8]	0.000	35.8 [34.2, 37.2]	0.004	36.2 [35.2, 37.4]	0.071
**C. Snow (Ins +)**	36.5 [36.0, 37.5]	12.5 [8.9, 26.8]	0.000	34.3 [32.3, 35.5]	0.000	35.4 [34.4, 36.7]	0.002
**D. Water (Ins +)**	36.5 [36.0, 37.5]	15.4 [10.2, 23.8]	0.000	34.5 [32.2, 36.4]	0.000	35.5 [33.9, 36.7]	0.000
