# Supplementary material for: The impact of environmental factors in pre-hospital thermistor-based tympanic temperature measurement: a pilot field study
Source: Scand J Trauma Resusc Emerg Med. 2015 Sep 24;23:72. doi: 10.1186/s13049-015-0148-5 (PMC4581419; doi:10.1186/s13049-015-0148-5)
Supplement: Additional file 2: — Concordance correlation coefficients for Δ Trect-tymp. (PDF 53 kb) [file 13049_2015_148_MOESM2_ESM.pdf]

Additional file 2

Concordance correlation coefficients for $\Delta \boldsymbol{T}_{rect\text{-}tymp}$

	**Baseline**	**0 min**	**5 min**	**10 min**
**A. Wind (ins-)**	0,0523	-0,0044	0,0088	-0,0021
**B. Wind (ins+)**	0,0461	0,0008	-0,0190	-0,0114
**C. Snow (ins+)**	0,0627	0,0018	0,0165	0,0367
**D. Water (ins+)**	0,0505	0,0007	0,0030	0,0184
